# Green synthesis of silver and iron nanoparticles of isolated proanthocyanidin: its characterization, antioxidant, antimicrobial, and cytotoxic activities against COLO320DM and HT29

**DOI:** 10.1186/s43141-020-00058-2

**Published:** 2020-08-20

**Authors:** Kiran P. Shejawal, Dheeraj S. Randive, Somnath D. Bhinge, Mangesh A. Bhutkar, Ganesh H. Wadkar, Namdeo R. Jadhav

**Affiliations:** 1 Department of Pharmaceutics, Rajarambapu College of Pharmacy, Kasegaon, Walwa, Sangli, Maharashtra 415404 India; 2 Department of Pharmaceutical Chemistry, Rajarambapu College of Pharmacy, Kasegaon, Walwa, Sangli, Maharashtra 415404 India; 3 Department of Pharmaceutics, Bharati Vidyapeeth College of Pharmacy, Kolhapur, Maharashtra 416013 India

**Keywords:** Proanthocynidin, Silver and iron nanoparticles, Antioxidant activity, Cytotoxicity, Colorectal cancer, COLO320DM, HT29

## Abstract

**Background:**

In the current research, we have developed silver and iron nanoparticles of isolated proanthocynidin (PAC) from grape seed by green synthesis and evaluated for antimicrobial, antioxidant activity and in vitro cytotoxicity against colon cancer cell lines.

**Results:**

One percent solution of isolated proanthocynidin in water was vigorously mixed with 1% silver nitrate and 1% ferric chloride solution and kept for 4 h, to yield PACAgNP and PACFeNP. The synthesized nanoparticles were characterized by UV, FTIR, XRD, and SEM analysis and evaluated for antimicrobial potential against selected microbes. Moreover, the synthesized nanoparticles were studied for DPPH assay and in vitro cytotoxicity using colon cancer cell lines COLO320DM and HT29 (MTT, SRB, and Trypan blue assay). UV spectroscopy confirmed the development of nanoparticles. SEM analysis showed that the particles were aggregated in the size range of 50 to 100 nm. Antimicrobial potential was found to be less against *Staphylococcus aureus*, *Pseudomonas aeruginosa*, and *Escherichia coli*, whereas cytotoxicity of PACAgNP and PACFeNP against COLO320DM and HT29 exhibited promising results as compared to the pure PAC. PACAgNP and PACFeNP exhibited 20.83 ± 0.33% and 18.06 ± 0.60% inhibition, respectively, against DPPH radical, whereas pure PAC showed 16.79 ± 0.32% inhibition and standard (ascorbic acid) exhibited 98.73 ± 0.18% inhibition of DPPH radical.

**Conclusion:**

The silver and iron nanoparticles were successfully developed by green synthesis method using isolated proanthocynidin which is economical and eco-friendly. The use of metal nanoparticles may open up a new opportunity for anticancer therapies to minimize the toxic effects of available anticancer drugs specifically in targeting specific site.

**Graphical abstract:**

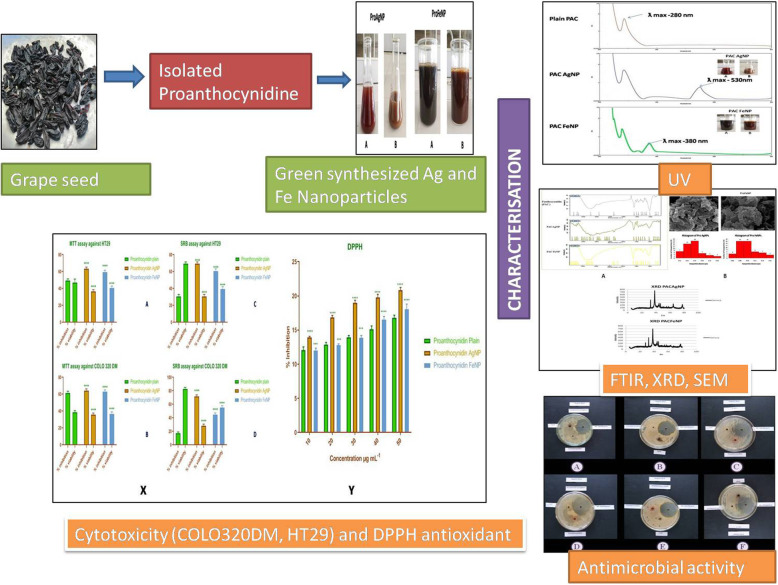

## Highlights

- The present research work is focused on green synthesis of silver and iron nanoparticles of isolated proanthocynidin from grape seed extract.
- This is the first attempt to assess scientifically the anticancer efficacy of the developed metal nanoparticles on in vitro colon cell lines HT29 and COLO320DM.
- The results of the study revealed that developed nanoparticles are having significant cytotoxic activity against colon cancer as compared with pure proanthocynidin (phytoconstituents).
- This is an eco-friendly, economical, easy, and rapid method of development of metal nanoparticles with minimum usage of hazardous chemicals.
- Use of isolated phytoconstituents which are devoid of toxic effects of all other anticancer drugs and better absorption as well as cellular uptake due to nanosize for targeting specific site will be a newer research area.

## Background

A foremost reason of cancer death in men and women is colorectal cancer (CRC), and it affects nearly 1 million people throughout the world every year [1]. It is the third most frequently diagnosed cancer in both men and women. About 103,170 new cases and around 51,700 deaths have been estimated to have occurred in 2012 in the USA. The occurrence of CRC in China is lower than that of western countries, but it has increased in the current years and has become a substantial cancer burden in China, mainly in the more developed areas. Epidemiological literature has shown that the daily consumption of fresh fruits and vegetables is connected with a reduced risk of cancer. The special effects may be moderately attributed to the presence of several polyphenolic compounds, which are known to exhibit antioxidant and free radical-scavenging properties [2].

It is very essential to deliver anticancer drug at the site of action, and the dosage form must exhibit high cell penetration or permeability and drug solubility. Nanoparticles may enter the human body via several routes. The probability of penetration depends on the size and surface properties of particles and on the anatomical structure of the specific sites of the exposure routes [3].

Nanotechnology can be termed as the exploitation of matter through specific chemical and/or physical processes to produce materials with specific properties, which can be utilized in particular applications [4, 5]. A nanoparticle can be a microscopic particle or material that has at least one dimension less than 100 nm in size [6–8]. Different bulk materials exhibit sole optical, electrical, thermal, physical, and chemical properties [9], and hence, they find a range of applications in the areas of environment, medicine, chemistry, energy, agriculture, information, communication, consumer goods, and heavy industry [10, 11]. Recently, there has been a keen interest in the green synthesis of nanoparticles [12].

Owing to the characteristic catalytic and optical properties of the metal nanoparticles as compared to the bulk material, they have fetched greater attention to date in multidisciplinary scientific areas especially in the pharmaceutical, and cosmetic, which position onward growth in commercial interest, and calling for effectual synthesis procedures to match the growing demand of silver, iron, and gold nanoparticles [13].

Ayurvedic and herbal formulations available in the market diverge in quality and therapeutic efficiency owing to the differences in composition of the plant phytoconstituents [14–16]. Advancement in the Ayurvedic herbal medicine has been revolutionized from the showing of phytochemicals and pharmacological activities to elucidating their mechanisms of action and sites of action [17]. Also currently, there is an increased interest in the herbal drugs and remedies for the treatment of chronic diseases [18].

Proanthocyanidins are the naturally available polyphenolic compound(s) which varied in chemical structure, pharmacological action, and characteristics and extensively available in fruits, seeds, vegetables, nuts, flowers, and bark [19].

Grape seed is an abundant source of proanthocyanidin (PACs), which is known to be a powerful inhibitor of aromatase activity; it is an enzyme expressed in higher levels in cancerous than in usual breast tissues [20]. PACs belong to a bigger class of abundant, plant-derived compounds, flavonoids, which offers numerous valuable health effects, largely because of its antioxidant properties [21, 22]. It has been shown to defend against oxidative stress and tobacco-induced DNA damage, and exhibited selective prominent cytotoxicity against some human cancers, including colon, lung, breast, prostate, and gastric carcinomas [23–28].

They are secondary plant metabolites available in many diverse kinds of fruits, vegetables, and plant-based beverages, and also available in cocoa, apple, grapes, tea, and red wine. PAC classes of condensed tannins are oligomers and polymers of (+)-catechin and (−)-epicatechin and other related flavonoids, chiefly linked by either B-type (C4 → C6 or C8) or A-type linkages (C2 → O7). Grape seed extract (GSE) falls into the B-type category, and grape seed extract-rich diets have been connected with a reduced risk of chronic cardiac diseases [29] and also a variety of common cancers, including colorectal cancer [30–32].

Proanthocyanins are strong free radical scavengers and are supposed to be contributors to the health benefits of fruits and vegetables [33, 34]. Proanthocyanidins obtained from grape seeds have been proven to protect against UV light-induced carcinogenesis, stop immune suppression, enhance interleukin (IL)-12, and decrease IL-10 [35]. Apple PACs have established synergistic effects with lysosomotropic compounds in increasing the anticancer properties targeting human colon cancer-derived metastatic cells [36, 37].

An environmentally friendly option is to prepare nanoparticles dependent on three important parameters, namely solvent medium, reducing and stabilizing or capping agent for NPs [38]. Therefore, the present study is important with respect to the development of metal nanoparticles of phytoconstituent specifically for targeting the cancer. It has many advantages like being eco-friendly and cost-effective, and mainly, the isolated phytoconstituents have prominent activity as compared with extract of plants and their parts; moreover, the side effects of the synthetic drugs can be avoided. Further, in vivo animal study of the metal nanoparticle-based formulation is the new area of research.

## Methods

### Chemicals

Proanthocynidin (PAC) was *obtained as a gift sample from Influx Healthcare, Mumbai, Maharashtra*. All the chemicals used in the study were of analytical grade. *Silver nitrate (AgNO*_*3*_*) and ferric chloride (FeCl*_*3*_*) were purchased from Loba Chem, Kolhapur.* Ciprofloxacin was obtained from Okasa Pharmaceutical, Satara. Cell line COLO320DM and HT29 were procured from NCCS, Pune, Maharashtra.

### Microorganism used

The test organisms used in this study were *Staphylococcus aureus* (ATCC 6538), *Pseudomonas aeruginosa*, (ATCC 10145), and *Escherichia coli* (ATCC 8739). The culture was obtained from Yashawantrao Chavan College of Science, Saidapur, Karad (MS), India—415110.

### Synthesis of metallic nanoparticles

#### Synthesis of proanthocyanidin silver nanoparticles (PACAgNPs)

**Fig. 1:**
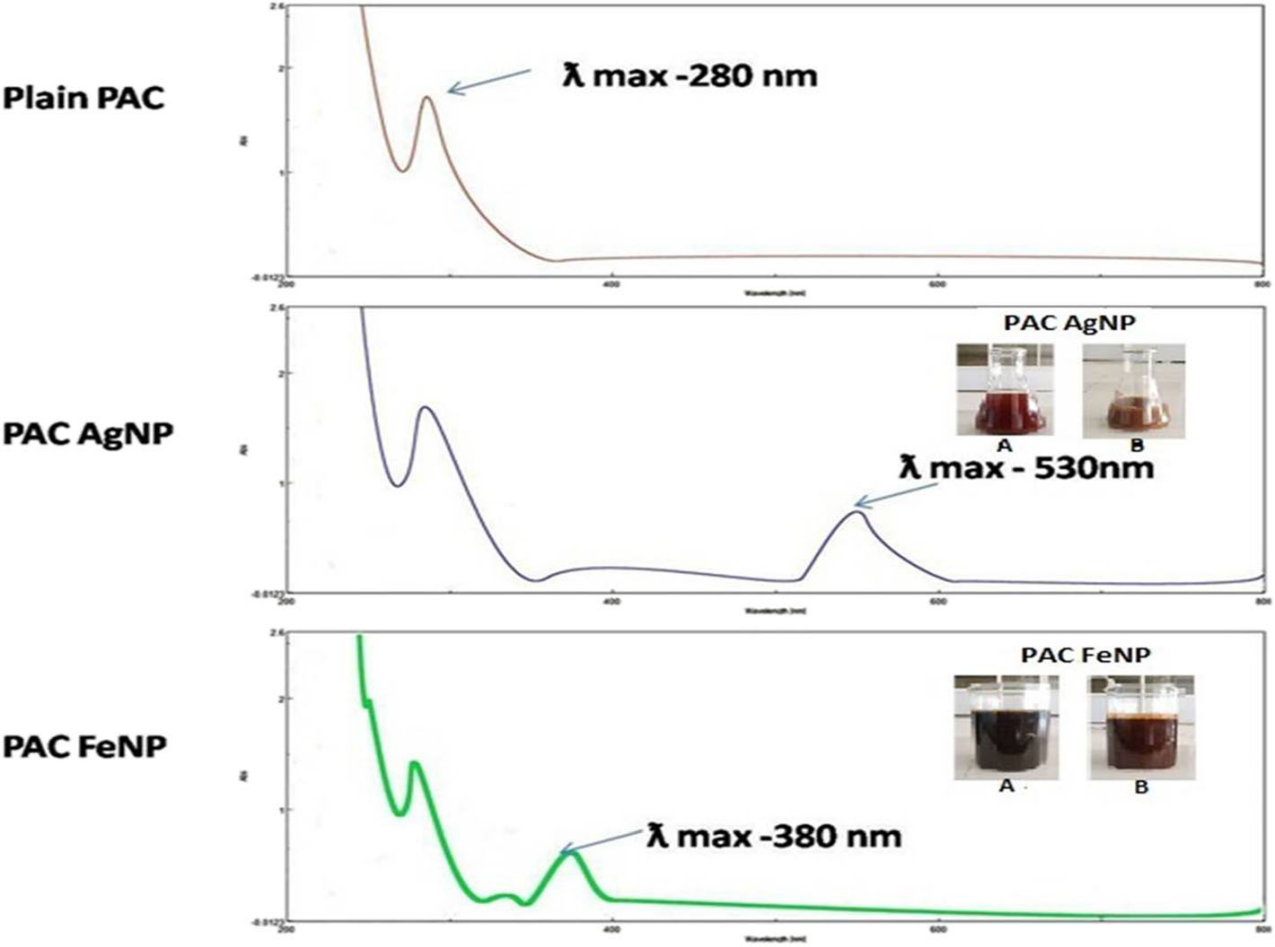
UV and synthesis NP

PACAgNPs were synthesized using proanthocyanidin solution and AgNO_3_ solution in accordance with the procedure mentioned by Phull et al. with minor modification [39]. Equal volumes (1:1) of 1% proanthocyanidin aqueous solution and 1% silver nitrate solution were incubated at ambient temperature for 2–3 h to obtain PACAgNPs. Synthesis of PACAgNPs was detected by naked eye with a change of color from dark red to faint brown, which was confirmed by UV spectroscopy. The collected PACAgNPs were centrifuged for 10 min at 10,000 rpm and dried in vacuum chamber at 35 °C shown in Fig. 1.

#### Synthesis of proanthocyanidin iron nanoparticles (PACFeNPs)

For the synthesis of ProFeNPs, proanthocyanidin aqueous solution and ferric chloride solution were used in accordance with the procedure mentioned by Raju et al. [40] with minor modification. Ferric chloride (1%) and proanthocyanidin solution (1%) were vigorously combined in a ratio of 1:1. The mixture was then placed on an orbital shaker for 24 h at an ambient temperature to obtain PACFeNPs. The synthesis of PACFeNPs was observed with a color change from dark black to dark brown, which was also confirmed by UV spectroscopy. The collected PACFeNPs were centrifuged for 10 min at 10,000 rpm and dried in vacuum chamber at 35 °C shown in Fig. 1.

### Characterization of nanoparticles

#### UV-Vis absorbance of PAC, PACAgNP, and PACFeNP

The development of metal nanoparticles of PACAgNP and PACFeNP by the proanthrocyanidin was recorded periodically using a UV spectrophotometer (Shimadzu). The samples were diluted with 2 mL of deionized water and measured for UV-Vis spectrum after the formation of nanoparticles that change in color. Deionized water was used as a blank for background correction. All samples were scanned from 200 to 800 nm.

#### SEM and histogram analysis of PACAgNP and PACFeNP

Scanning electron microscopy (SEM) is a normally used method of evaluation and morphological analysis of nanoparticles at the nanometer to micrometer scale. Developed PACAgNP and PACFeNP were characterized using a high-resolution scanning electron microscope (Schottky field emission scanning microscope SU5000). The samples were prepared by a simple drop coating of suspended gold solution on to an electric clean glass and allowed the solvent to evaporate, and the samples were dried at room temperature and analyzed in a microscope.

#### FTIR spectroscopy analysis of PAC, PACAgNP, and PACFeNP

To recognize the different biomolecules present in the proanthocynidin and the phytocompounds in Ag and Fe, nanoparticles after synthesis were analyzed by FTIR (Bruker Alpha Echo ATR). Spectrum was recorded in the range of 400–4000 cm^−1^.

#### XRD analysis of PACAgNP and PACFeNP

The green synthesized silver and iron nanoparticles of PAC were evaluated by XRD analysis using an XRD-6000 X-ray diffractometer (Bruker D8 discover) operated at a voltage of 40 kV and 30 mA with Cu Kα radiation in θ–2θ configurations. The crystalline size was determined from the width of the XRD peaks by assuming that they were free from non-uniform strains.

### Antimicrobial activity of PAC, PACAgNP, and PACFeNP

In vitro antimicrobial activity was performed using the agar well diffusion technique [41]. The sterile agar was inoculated with the bacterial culture (*S. aureus*, *P. aeruginosa*, and *E. coli*) for 48 h at 37 °C. Antimicrobial activities were tested on nutrient medium against *S. aureus*, *P. aeruginosa*, and *E. coli*, which are representative types of Gram-positive and Gram-negative organisms. Wells were bored by using a sterile borer. Standard solution (ciprofloxacin) and test samples PAC, PACAgNP, and PACFeNP (5 mg/mL was prepared by dissolving the test sample in DMSO) were placed into the wells (80 μL). Plates were then kept for 2 h in the refrigerator to enable prediffusion of the extracts into the agar. Finally, the plates were incubated overnight (24 h at 37 °C.) The antimicrobial activity was determined by measuring the diameter of zone of inhibition [42, 43].

### In vitro cytotoxicity studies of PAC, PACAgNP, and PACFeNP by using MTT assay

Human HT29 cell and COLO320DM were obtained from the National Center for Cell Sciences, Pune, MS, India, 411007. The cell cultures were maintained in DMEM supplemented with 10% fetal bovine serum. The cells were plated at a density of 1 × 1 cells per well in a 96-well plate and cultured for 24 h at 37 °C. The cells were subsequently exposed, the plates were incubated for 24 h, and cell proliferation was measured by adding 10 μL of MTT (thiazolyl blue tetrazolium bromide) dye (5 mg/mL in phosphate-buffered saline) per well. The plates were incubated for a further 4 h at 37 °C in a humidified chamber containing 5% CO_2_. Formazan crystals formed due to reduction of dye by viable cells in each well were dissolved in 200 μL DMSO, and absorbance was read at 490 nm.

Finally, the percent cytotoxicity of the compounds was calculated by using the following formula:

$$ \mathbf{Percent}\ \mathbf{Cytotoxicity}=\frac{\mathrm{Reading}\ \mathrm{of}\ \mathrm{control}\hbox{-} \mathrm{Reading}\ \mathrm{of}\ \mathrm{treated}\ \mathrm{cells}}{\mathrm{Reading}\ \mathrm{of}\ \mathrm{control}}\times 100 $$

Since the absorbance was directly associated with the number of viable cells, the percent viability was determined from the absorbance.

### In vitro cytotoxicity studies of PAC, PACAgNP, and PACFeNP by using SRB assay

Human HT29 cells and COLO320DM were maintained in DMEM supplemented with 10% fetal bovine serum. The cells were plated at a density of 1 × 10^4^ cells per well in a 96-well plate and cultured for 24 h at 37 °C. The cells were subsequently exposed to 100 μg/mL compound. After drug incubation, 50 μL TCA (50%) was kept for 1 h at 4 °C. Then, the plate was washed with TDW (triple distilled water) and air dried. Thereafter, 100 μL SRB dye was added in each well and kept for 30 min at room temperature. Again, the plate was washed three times with 1% acetic acid and air dried. Finally, 200 μL Tris buffer was added, and the absorbance was read at 490 nm.

The percent cytotoxicity of the compounds was calculated by using following formula:

$$ \mathbf{Percent}\ \mathbf{Cytotoxicity}=\frac{\mathrm{Reading}\ \mathrm{of}\ \mathrm{control}\hbox{-} \mathrm{Reading}\ \mathrm{of}\ \mathrm{treated}\ \mathrm{cells}}{\mathrm{Reading}\ \mathrm{of}\ \mathrm{control}}\times 100 $$

### In vitro cytotoxicity studies of PAC, PACAgNP, and PACFeNP by using Trypan blue assay

The dye exclusion test is used to find out the number of viable cells present in a cell suspension. It is based on the principle that live cells possess undamaged cell membranes that exclude certain dyes, such as Trypan blue, eosin, or propidium, whereas dead cells do not exclude. In this test, a cell suspension is simply mixed with Trypan blue dye and then visually examined to determine whether cells take up or exclude dye. In the study presented here, a viable cell will have undamaged a clear cytoplasm whereas a non-viable cell will have a blue cytoplasm.

Fifty microliters of cell lines of human HT29 cells and Colo 320 D was taken in micro-centrifuge tube. They were incubated for 3 min and then added 50 μL of all samples of nanoparticles in concentration of 100 μg mL^−1^ which were prepared by dissolving in phosphate buffer pH 7.4 and DMSO. They were incubated in CO_2_ incubator for 3 min, and thereafter, Trypan blue (0.4%) 50 μL was added in each tube. They were further incubated for 3 min in CO_2_ incubator and analyzed for total viable cells and non-viable cells by using Nubars slide [44].

### Antioxidant activity of PAC, PACAgNP, and PACFeNP, by DPPH (2,2-diphenyl-2-picryl hydrazyl hydrate) assay

The scavenging ability of PAC, PACAgNP, and PACFeNP on the stable free radical was calculated with the method expressed by Mensor et al. [45]. Twenty microliters of PAC, PACAgNP, and PACFeNP solutions was separately added in three labeled test tubes. Subsequently, 0.5 mL of methanolic solution of DPPH and 0.48 mL of methanol were added to each test tube, after which all tubes were allowed to react at an ambient temperature for 30 min. The control was prepared as described above, without any extract and nanoparticles. Methanol was used to correct the baseline. After 30 min of incubation, the discoloration of the purple color was measured under a UV-Visible spectrophotometer. The radical-scavenging activity was determined by the following formula [45]:

$$ \mathbf{Scavenging}\ \mathbf{activity}\ \left(\%\right)=\frac{{\mathrm{A}}_{\mathrm{abs}}\ \left(\mathrm{Control}\right)\hbox{-} {\mathrm{A}}_{\mathrm{abs}}\left(\mathrm{Sample}\right)}{{\mathrm{A}}_{\mathrm{abs}}\ \left(\mathrm{Control}\right)}\times 100 $$

where A (control) is the absorbance of control sample PACAgNP and PACFeNP measured at 530 nm and 275 nm, respectively, and A (control) is the absorbance of PAC measured at 280 nm.

### Statistical analysis

Statistical data of the cytotoxicity were assessed on GraphPad Prism 8 for Windows 64 bit with version 8.0.1 (244). Results were analyzed by one-way ANOVA with Dunnett’s post-test analysis of variance. The mean standard error mean (SEM) of all calculated values was shown in each group. A value of *P* < 0.05, 0.01, or 0.001 was considered statistically significant.

## Results

### UV-Vis spectroscopy of PAC, PACAgNP, and PACFeNP

When the aqueous PAC was mixed with aqueous AgNO_3_ and FeCl_3_ solution, the color of the solution changed which indicated the formation of silver and iron nanoparticles. This change in color was due to the collective coherent oscillation of conduction electrons at the surface of the nanoparticles that interact with the oscillating electric field of the incident light, a phenomenon called surface plasmon resonance (SPR). This change in color indicated the reduction of Ag and Fe ions which was traced with UV-Vis spectroscopy. The PAC showed ƛ max at 280 nm, and silver nanoparticle PACAgNP possesses specific wavelength that can absorb at ƛ max 530 nm, whereas iron nanoparticles PACFeNP exhibit ƛ max at 380 nm as shown in Fig. 1.

### FTIR spectrum of PAC, PACAgNP, and PACFeNP

**Fig. 2:**
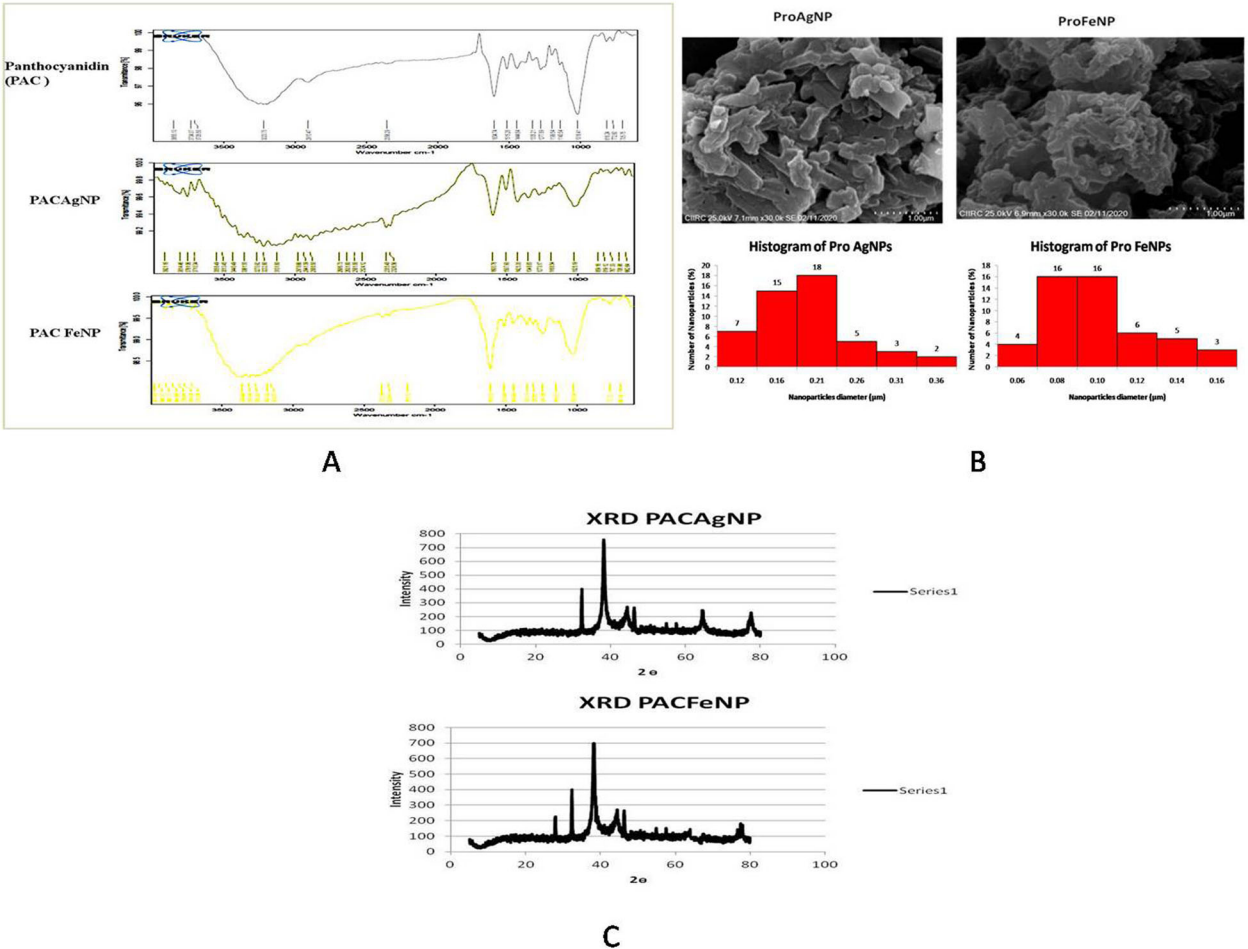
**a** FTIR. **b** SEM histogram. **c** XRD

In the FTIR of proanthocynidin, fairly sharp peaks at 3223.75, 2910.47, 1604.74, 1515.78, 1444.64, 1217.89, and 1018.41 cm^−1^ were observed, which indicate the presence of the functional group present in the compound. However, the aromatic at 1604.74 (C-C-valence) cm^−1^, the OH phenolic at 3223.75 (O-H-valence) and 2910.47 (C-H-valence, arene), alkane (OCH_3_), the methoxylic at 1217.89 cm^−1^, and C-O stretching at 1044.64 appeared in the IR spectrum of complex as shown in Fig. 2a.

#### PACAgNP

These characteristic vibrations after reduction of Ag+ ions were shifted to new peaks at 3222.80, 2888.50, 1600.76, 1507.93, 1425.35, 1188.54, and 1025.79 cm^−1^, which indicated the presence of the functional group present in the compound. However, the aromatic at 1600.76 (C-C-valence) cm^−1^, the OH phenolic at 3222.80 (O-H-valence) and 2888.50 (C-H-valence, arene), alkane (OCH_3_), the methoxylic at 1188.54 cm^−1^, and C-O stretching at 1025.79 appeared in the IR spectrum of complex as shown in Fig. 2a.

In addition, bio-reduction showed that the 3222.80, 2888.50, and 1025.79 cm^−1^ bands were suppressed in the AgNP. Proanthocynidin and NPs showed similar absorption bands, indicating that NPs might be stabilized by proanthocynidin. On the basis of the orange, yellow, and brownish green color of the biomass and the groups suggested by FTIR analysis, it was hypothesized that proanthocynidin may be involved in silver nanoparticle synthesis.

#### PACFeNP

These characteristic vibrations after reduction of Fe3+ ions were shifted to new peaks at 3310.40, 2900.05, 1610.53, 1513.64, 1448.60, 1149.07, and 1025.38 cm^−1^, which indicated the presence of the functional group present in the compound. However, the aromatic at 1610.53 (C-C-valence) cm^−1^, the OH phenolic at 3310.40 (O-H-valence) and 2900.05 (C-H-valence, arene), alkane (OCH_3_), the methoxylic at 1149.07 cm^−1^, and C-O stretching at 1025.38 appear in the IR spectrum of complex as shown in Fig. 2a. In addition, bio-reduction showed that the 3310.40, 2900.05, and 1149.07 cm^−1^ bands were suppressed in the FeNP. Proanthocynidin and NPs showed similar absorption bands, indicating that NPs might be stabilized by proanthocynidin. On the basis of the orange, yellow, and blackish color of the biomass and the groups suggested by FTIR analysis, it was hypothesized that proanthocynidin may be involved in iron nanoparticle synthesis.

#### SEM and histogram analysis of PACAgNP and PACFeNP

A scanning electron microscope was used to analyze the structure of PACAgNP and PACFeNP nanoparticles that are developed and represented in Fig. 2b. The nanoparticles formed were aggregated having a size range of 100 to 120 nm. This aggregation of the nanoparticles can be minimized or prohibited by increasing the concentration of the proanthocynidin extract. Histograms of both the nanoparticles have been represented in Fig. 2b exhibiting average particle size PACFeNP 0.09083 + 0.02627 μm and PACAgNP 0.17746 + 0.05784 μm.

#### XRD spectrum of PACAgNP and PACFeNP

The XRD pattern of the synthesized silver and iron nanoparticles formed using proanthocynidin is shown in Fig. 2c. The diffraction peak at 2θ = 38° and subsequent higher order reflections can be indexed to the Ag and other facets of silver nanoparticles by comparing JCPDS file no: 89-3722; in case of PACFeNP, the peak shown at 2θ = 33° corresponds to the iron compared with standard XRD for iron (JCPDS data: pdf no 39:1346).

The XRD spectrum also revealed a weak peak around 2θ = 30°, which can be attributed to the phytochemical components. It, thus, confirmed that the nanoparticles formed on the membrane consisted of crystalline. XRD indicated possible multicomponent product formation at higher energy.

#### Antibacterial activity of PAC, PACAgNP, and PACFeNP

**Fig. 3:**
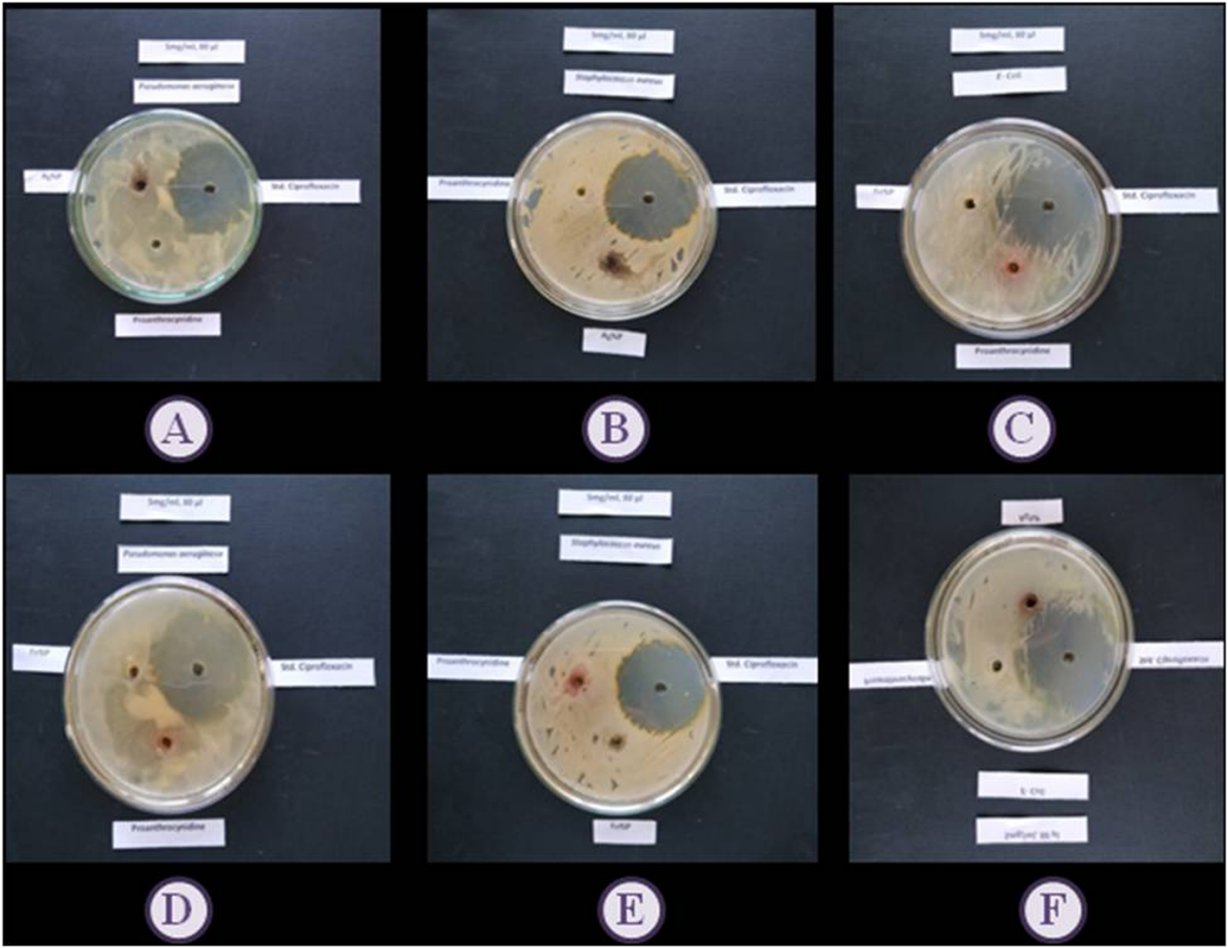
Antimicrobial activity

The zones of inhibition revealed that there is very little antimicrobial potential of PAC, PACAgNP, and PACFeNP against *Pseudomonas aeruginosa*, *Staphylococcus aureus*, and *E coli.* The results are highlighted in Table 1, and the zone of inhibition is depicted in Fig. 3.

**Table 1:** Results of antibacterial activity of PAC, PACAgNP, and PACFeNP against selected microbial strains

Sr. no.	Sample name	Zone of inhibition diameter (mm) against the selected microorganisms		
		*Pseudomonas aeruginosa*	*Staphylococcus aureus*	*E. coli*
1	PAC pure	6.00 ± 0.10	0.0	0.00
2	PACAgNP	7.0 ± 0.11	12.00 ± 0.16	6.00 ± 0.13
3	PACFeNP	6.0 ± 0.14	6.0 ± 0.12	0.00
4	Ciprofloxacin std.	35 ± 0.11	38 ± 0.13	40 ± 0.12

Values are expressed in triplicate mean SD

#### Results of cytotoxicity of PAC, PACAgNP, and PACFeNP

**Fig. 4:**
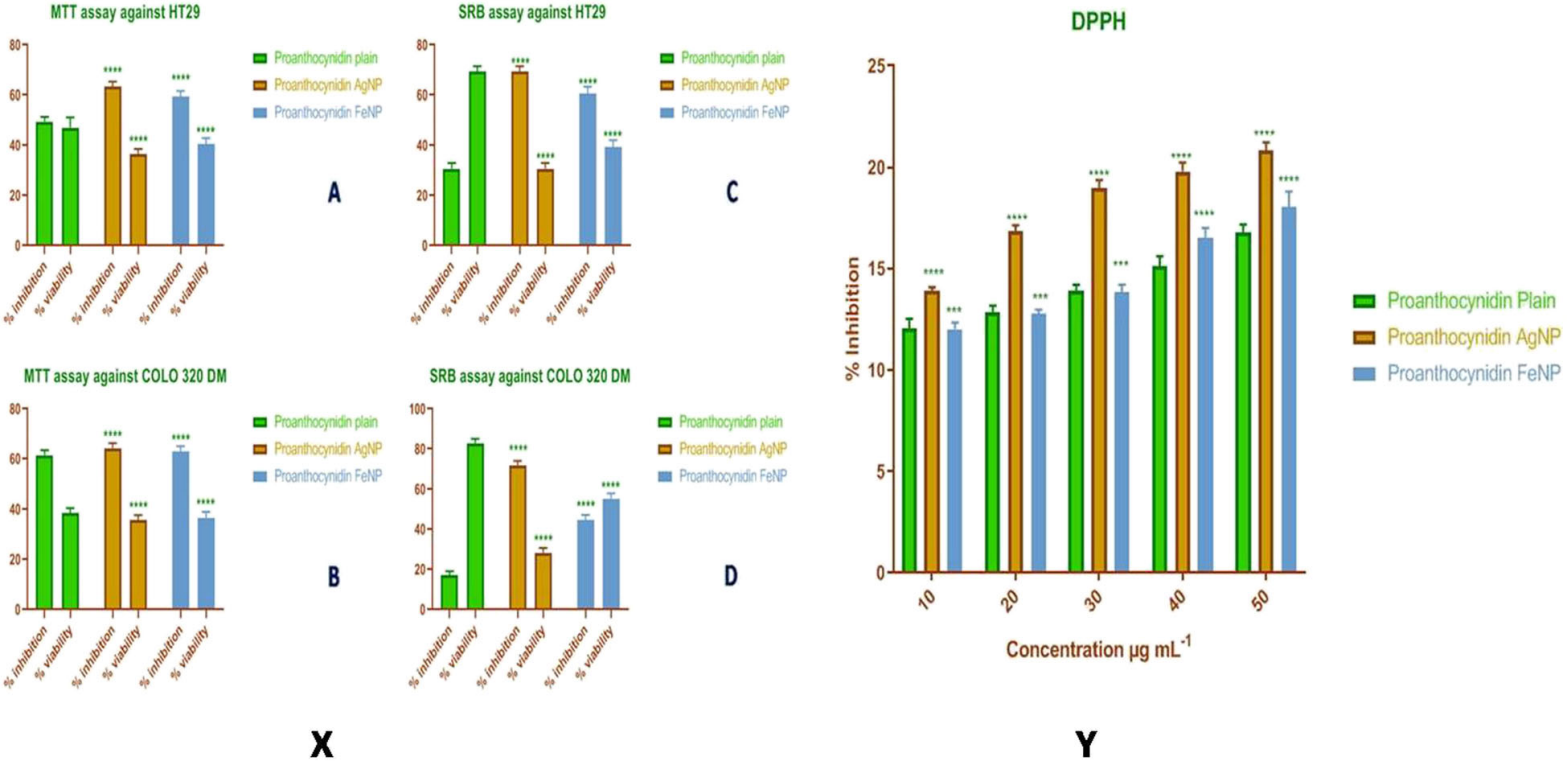
Graph of **x** cytotoxicity and **y** antioxidant DPPH

The results of cytotoxicity assay by MTT and SRB assay have been presented in Table 2 with respect to two different colon cancer cell lines namely COLO320DM and HT29. A variation in the results of different assay was observed; however, the silver nanoparticles exhibited better activity than pure PAC in both the methods. The results of MTT assay against HT29 demonstrated that PACAgNP showed maximum 63.34 ± 1.64% inhibition represented in Fig. 4x (A). In case of COLO320DM, silver nanoparticles exhibited 64.27 ± 1.63% inhibition as represented in Fig. 4x (B), whereas the SRB assay of PAC, PACAgNP, and PACFeNP against HT29 revealed 69.21 ± 1.86% inhibition by PACAgNP as shown in Fig. 4x (C). In case of COLO320DM SRB assay, a maximum of 71.6 ± 1.97% inhibition was exhibited by silver nanoparticles as depicted in Fig. 4x (D).

**Table 2:** Results of cytotoxicity of PAC, PACAgNPs, and PACFeNP by MTT and SRB assay using COLO320DM and HT29 cell lines

Compound	Mean OD	Percent inhibition	Percent viability
MTT assay against HT29 (control—0.321)			
Proanthocynidin plain	0.161	49.53 ± 1.54	47.13 ± 3.32
Proanthocynidin AgNP	0.117	63.34 ± 1.64	36.65 ± 1.64
Proanthocynidin FeNP	0.129	59.39 ± 1.84	40.60 ± 1.84
MTT assay against COLO320DM (control—0.384)			
Proanthocynidin plain	0.146	61.49 ± 1.66	35.51 ± 1.66
Proanthocynidin AgNP	0.137	64.27 ± 1.63	35.72 ± 1.63
Proanthocynidin FeNP	0.142	63.17 ± 1.63	36.49 ± 2.07
SRB assay against HT29 (control—0.292)			
Proanthocynidin plain	0.204	30.71 ± 1.88	69.29 ± 1.88
Proanthocynidin AgNP	0.090	69.21 ± 1.86	30.78 ± 1.86
Proanthocynidin FeNP	0.115	60.57 ± 2.20	39.43 ± 2.20
Proanthocynidin plain	0.204	30.71 ± 1.88	69.29 ± 1.88
SRB assay against COLO320DM (control—0.252)			
Proanthocynidin plain	0.209	17.11 ± 1.72	82.89 ± 1.72
Proanthocynidin AgNP	0.073	71.6 ± 1.97	28.4 ± 1.97
Proanthocynidin FeNP	0.141	44.60 ± 2.18	55.39 ± 2.18

Values are expressed in triplicate mean SD

The results of Trypan blue assay revealed that silver nanopaticles PACAgNP showed 85.63 ± 0.27% non-viability of COLO320DM cell line when observed on a Motic microscope, whereas iron nanoparticles PACFeNP exhibited 94.57 ± 0.36% inhibition of HT29 cell lines as represented in Table 3.

**Table 3:** Results of cytotoxicity of PAC, PACAgNPs, and PACFeNP by using Trypan blue assay

Sr. no	Drug	COLO320DM		HT29	
		Percent viability	Percent non-viable	Percent viability	Percent non-viable
1	Proanthocynidin plain	31.45 ± 0.49	68.88 ± 0.36	25.48 ± 0.31	74.70 ± 0.58
2	Proanthocynidin AgNP	14.89 ± 0.30	85.63 ± 0.27	9.56 ± 0.26	90.23 ± 0.43
3	Proanthocynidin FeNP	25.7 ± 0.42	74.5 ± 0.28	5.34 ± 0.18	94.57 ± 0.36

Values are expressed in triplicate mean SD

#### Results of DPPH (2,2-diphenyl-2-picryl hydrazyl hydrate) assay of PAC, PACAuNP, and PACFeNP

The DPPH activity results demonstrated effective free radical percent scavenging potential of PAC, PACAuNP, and PACFeNP as depicted in Fig. 4y. As compared to ascorbic acid (standard), the concentration response curves of DPPH radical-scavenging activity of PAC, PACAuNP, and PACFeNP are shown in Table 4. It was observed that PACAgNPs were more effective than PAC extract and PACFeNPs. At a concentration of 50 μg mL^−1^, the scavenging activity of PACAgNPs was observed to be 20.83 ± 0.33%, while at similar concentration, for the PACFeNPs it was reported to be 18.06 ± 0.60%. The outcome of antioxidants on DPPH is thought to be due to their hydrogen donating ability.

**Table 4:** Antioxidant activity of DPPH radical-scavenging activity

Concentrations, μg mL^−1^	Standard, percent inhibition	PAC, percent inhibition	PACAgNPs, percent inhibition	PACFeNPs, percent inhibition
10	93.29 ± 0.60	12.07 ± 0.37	13.92 ± 0.13	12.01 ± 0.27
20	95.13 ± 0.54	12.86 ± 0.25	16.83 ± 0.24	12.78 ± 0.16
30	96.16 ± 0.17	13.89 ± 0.24	18.95 ± 0.33	13.83 ± 0.30
40	97.76 ± 0.29	15.11 ± 0.41	19.80 ± 0.35	16.52 ± 0.40
50	98.73 ± 0.18	16.79 ± 0.32	20.83 ± 0.33	18.06 ± 0.60
IC50	–	12.50 ± 0.30	7.37 ± 0.27	9.31 ± 0.22

Values are expressed in triplicate mean SD

## Discussion

Engineered nanomaterials showed imperative benefits due to their unique nanostructure, along with their significant properties for the designed applications [46]. Metallic NPs have been synthesized using several different methods such as chemical reduction, electrochemical, microbiological reduction, ultrasonication method, and microwave radiation [47]. The present research work is focused on green synthesis of silver and iron nanoparticles of isolated proanthocynidin from grape seed extract. It is an eco-friendly, economical, easy, and rapid method of development of metal nanoparticles with minimum usage of hazardous chemicals. The developed silver and iron nanoparticles were characterized by UV spectroscopy which showed the change in absorbance after development of nanoparticles. FTIR spectrum provides the information about the chemical change of the functional groups involved in bio-reduction [48, 49]. The FTIR spectra of developed nanoparticles confirmed the formation of silver and iron nanoparticles as characteristic vibrations after reduction of Ag+ ions were shifted to new peaks at 3222.80, 2888.50, 1600.76, 1507.93, 1425.35, 1188.54, and 1025.79 cm^−1^. And characteristic vibrations after reduction of Fe3+ ions were shifted to new peaks at 3310.40, 2900.05, 1610.53, 1513.64, 1448.60, 1149.07, and 1025.38 cm^−1^. Also, characteristic color change can be attributed to the surface plasmon resonance of deposited AgNPs. And it clearly indicated the development of nanoparticles [50].

The size of nanoparticles and its morphology were clearly observed in the SEM images. The average size for PACFeNP was observed to be 0.09083 + 0.02627 μm and 0.17746 + 0.05784 μm for PACAgNP. The XRD results revealed weak peak at 2θ = 30° for phytoconstituent, whereas diffraction peak at 2θ = 38° was observed for silver nanoparticles and iron nanoparticles showed the peak at 2θ = 33°.

In case of antimicrobial activity, the developed silver and iron nanoparticles exhibited little antimicrobial potential as compared to pure proanthocynidin. Metal NPs increase the antibacterial potential due to the formation of reactive oxygen species (ROS), developed from different types of iron oxide nanoparticles like FeO, Fe2O3, and Fe3O4. The free radical produced in the reaction causes intracellular stresses that can damage the DNA of the cell [51].

The in vitro cytotoxicity results against HT29 and COLO320DM showed that PACAgNP exhibited 63.34 ± 1.64% inhibition (MTT assay) and 69.21 ± 1.86% inhibition (SRB assay) against HT29. In case of COLO320DM, PACAgNP demonstrated 64.27 ± 1.63% inhibition (MTT assay) and 71.6 ± 1.97% inhibition (SRB assay).

In DPPH assay, PACFeNP and PACAgNP exhibited 18.06 ± 0.60% and 20.83 ± 0.33% inhibition, respectively. The obtained parameters of the characterization and evaluation of the nanoparticles clearly revealed that as compared to proanthocynidin, the silver and iron nanoparticles possess better antioxidant and anticancer potential against colorectal cancer. Thus, the use of isolated phytoconstituent(s) which are devoid of side effects and promoting better absorption and cellular uptake owing to their nanosize will certainly achieve effective targeting and has to be provided greater attention as a newer research area. Also, several review papers have been published about the synthesis of silver nanoparticles using natural polymers like k-Carrageenan and synthetic polymers like poly vinyl pyrrolidone and polyethylene glycol to improve the strength and stability of nanoparticles. As per the study of Moustafa, the use of natural and synthetic polymer for the development of silver nanoparticles opened up new research area in medicinal and biological field along with food industry [52].

## Conclusion

We have successfully synthesized PACAgNPs and PACFeNPs using proanthocynidin isolated from grape seed extract by employing a green synthesis method which is relatively simple and environmentally benign. PACAgNPs and PACFeNPs were obtained, with particles between 50 and 100 nm in size. It is easy and cost-effective and does not involve any harmful and poisonous chemicals. All the other characterization like UV, FTIR, XRD, and SEM confirmed the development of nanoparticles. We have observed significant antioxidant activity and free radical-scavenging capacities. PACAgNPs and PACFeNPs exhibited a little antibacterial activity against the selected strains of microbes. In a nutshell, the study showed that the developed NPs from the isolated PAC exhibit beneficial antioxidant and anticancer potential when assessed by three different in vitro assay methods with specifically colon cancer cell lines. Thus, it may open up a new opportunity for anticancer therapies that need further research.

## Data Availability

All the data required for the processing of the conclusions are presented in the “Results” section. Supporting data was included separately.

## Abbreviations

PAC: Proanthocynidin
PACAgNP: Proanthocynidin silver nanoparticle
PACFeNP: Proanthocynidin iron nanoparticle
UV: Ultraviolet visible spectroscopy
FTIR: Fourier transform infrared spectroscopy
XRD: X-ray diffraction
SEM: Scanning electron microscope
DMEM: Dulbecco’s modified Eagle’s medium
DPPH: 2,2-Diphenyl-2-picryl hydrazyl hydrate
MTT: 3-(4,5-Dimethylthiazol-2-yl)-2,5-diphenyltetrazolium bromide
SRB: Sulforhodamine B
